# Knee osteoarthritis patients with more subchondral cysts have altered tibial subchondral bone mineral density

**DOI:** 10.1186/s12891-018-2388-9

**Published:** 2019-01-05

**Authors:** Wadena D. Burnett, Saija A. Kontulainen, Christine E. McLennan, Diane Hazel, Carl Talmo, David R. Wilson, David J. Hunter, James D. Johnston

**Affiliations:** 1 University of Saskatchewan, Saskatoon, SK Canada; 2 New England Baptist Hospital, Boston, MA USA; 3 University of British Columbia, Vancouver, BC Canada; 4 University of Sydney, Sydney, NSW Australia; 5 Department of Mechanical Engineering, University of Saskatchewan, 57 Campus Dr, Saskatoon, SK S7N 1G9 Canada

**Keywords:** Osteoarthritis, Tibia, Bone mineral density, Computed tomography

## Abstract

**Background:**

Subchondral bone cysts are a widely observed, but poorly understood, feature in patients with knee osteoarthritis (OA). Clinical quantitative computed tomography (QCT) has the potential to characterize cysts in vivo but it is unclear which specific cyst parameters (e.g., number, size) are associated with clinical signs of OA, such as disease severity or pain. The objective of this study was to use QCT-based image-processing techniques to characterize subchondral tibial cysts in patients with knee OA and to explore relationships between proximal tibial subchondral cyst parameters and subchondral bone density as well as clinical characteristics of OA (alignment, joint space narrowing (JSN), OA severity, pain) in patients with knee OA.

**Methods:**

The preoperative knee of 42 knee arthroplasty patients was scanned using QCT. Patient characteristics were obtained, including OA severity, knee pain, JSN, and alignment. We used 3D image processing techniques to obtain cyst parameters including: cyst number, cyst number per proximal tibial volume, cyst volume per proximal tibial volume, as well as maximum and average cyst volume across the proximal tibia, as well as regional bone mineral density (BMD) excluding cysts. We used Spearman’s correlation coefficients to explore associations between patient characteristics and cyst parameters.

**Results:**

At both the medial and lateral compartments of the proximal tibia, greater cyst number and volume were associated with higher BMD. At the lateral region, cyst number and volume were also associated with lateral OA severity, lateral JSN, alignment and sex. Pain was not associated with any cyst parameters at any region.

**Conclusion:**

Cyst number and volume were associated with BMD at both the medial and lateral compartments. Lateral cyst number and volume were also associated with joint alignment, OA severity, JSN and sex. This is the first study to use clinical QCT to explore subchondral tibial cysts in patients with knee OA and provides further evidence of the relationships between subchondral cysts and clinical OA characteristics.

**Electronic supplementary material:**

The online version of this article (10.1186/s12891-018-2388-9) contains supplementary material, which is available to authorized users.

## Background

Knee osteoarthritis (OA) is a painful and debilitating disease characterized by cartilage deterioration and altered subchondral bone. Recently, there has been increasing interest in the role of subchondral bone cysts in OA progression; in particular how subchondral bone cysts may influence pain [1–3], or how subchondral bone cysts influence subchondral bone mechanical behaviour [4].

Subchondral cysts are typically spherical or ellipsoidal cavities within the subchondral bone region and are related to both altered subchondral bone and cartilage degeneration in patients with OA [5–7]. Recent studies have indicated associations between subchondral bone cysts and pain [8] as well as bone marrow lesions (BMLs) in patients with knee OA [6]; though, evidence of relationships between cysts and other patient characteristics (e.g., disease severity, joint space narrowing, alignment) is limited. A clear understanding of disease pathogenesis is crucial for rational therapeutic targeting [9], particularly understanding of which structures contribute to pain [9]. As subchondral cysts are related to OA progression [10, 11], it is meaningful to investigate associations between subchondral cyst parameters (e.g., number and size) and OA severity and related pain.

Subchondral cysts are associated with higher localized stress [4], which could stimulate bone remodelling or bone alterations. Ex vivo studies at both the hip using high-resolution computed tomography (HR-QCT) [5], and the tibia using micro-computed tomography (micro-CT) [7] report changes in bone mineral density (BMD), especially in regions adjacent to cysts. These ex vivo studies are able to evaluate cyst number and size, but are not able to correlate cyst properties with important clinical symptoms such as pain. Clinical techniques, such as magnetic resonance imaging (MRI) and quantitative computed tomography (QCT) have the potential to offer three-dimensional (3D) characterizations of cyst structure. Using MRI techniques, cysts can be distinguished from other bony features, such as BMLs [12, 13], but it is difficult to reliably quantify BMD. Clinical QCT has potential to characterize cysts in vivo, to explore relationships with clinical OA symptoms (such as pain), to determine tibial BMD, and could potentially be used to evaluate 3D cyst development throughout disease progression to determine the role of cysts in OA. However, it is unclear which specific cyst parameters (e.g., number, size) are associated with clinical symptoms, and which parameters are associated with BMD.

The objective of this study was to use QCT and image-processing techniques to determine relationships between subchondral cyst parameters and subchondral BMD as well as clinical characteristics of OA (OA severity, OA-related pain, alignment, joint space narrowing) in patients with knee OA.

## Methods

### Study participants

Forty-two participants (17 M: 25F; mean age 64, SD ± 10.2 years; mean BMI 28.6 ± 3.7; 18 L:24R) with OA were recruited before total knee replacement. Study exclusion criteria included: pregnant women, patients having a revision replacement instead of primary knee replacement, and patients with a prior history of bone pathology at the knee joint. The Institutional Research Board of the New England Baptist Hospital approved the study. Informed written consent was obtained from all study participants. Patient selection, inclusion, and exclusion was intentionally very broad due to the exploratory nature of this study, as well as limited availability of participants scheduled for knee replacement surgery at the participating hospital.

### Participant ASSESMENT

The participating orthopaedic surgeon (CT) used standardized radiographs with Kellgren-Lawrence (KL) scoring [14] to classify OA severity. The participating surgeon also used standardized radiographs to classify compartment-specific joint space narrowing (JSN) and osteophyte presence. A single researcher (WDB) used compartment-specific JSN, osteophyte scores and measures of sclerosis from serial CT images to classify compartment-specific OA severity. We also included CT-measured knee alignment in our analysis. Knee alignment was categorized as varus, neutral, or valgus and measured using coronal and sagittal CT reconstructions to determine an estimate of mechanical alignment [3]. In brief, medial and lateral joint space widths were evaluated at equal distances from the tibial spine permitting an estimation of alignment between the femoral and tibial axes. Neutral alignment was defined as 178° ± 2^o^ (i.e., 176-180^o^) [3]. We defined an angle less than 176^o^ as varus alignment and an angle greater than 180^o^ as valgus alignment. For this analysis, alignment was treated as a categorical variable, where participants exhibited either varus (− 1), neutral (0), or valgus (1) alignment. Pain severity was measured at the affected knee joint using the pain subsection of the Western Ontario McMasters Osteoarthritis Index (WOMAC) [15]. Participants were asked to assess the level of pain in the affected knee joint within the past 24-h while walking on a flat surface, going up or down stairs, lying down in bed at night, sitting or lying down, and standing upright using a 5-point Likert scale (0 to 4). Individual element pain scores were then summed for a possible total WOMAC pain score of 20, with scores ranging from 4 to 14. We also included pain lying down in bed at night (nocturnal pain), scored from 0 (none) to 4 (extreme) as a patient characteristic to correspond with our earlier work evaluating pain and subchondral BMD [2] We used the Self-Administered Comorbidity Questionnaire [16] to assess participants for any potential confounding comorbidities (e.g., diabetes, heart disease).

### QCT acquisition

We used a single energy clinical CT scanner (Lightspeed 4-slice, General Electric, Milwaukee, WI, USA) for bone imaging. A solid QCT spine reference phantom of known bone mineral densities (Model 3 T, Mindways Software Inc., Austin, TX, USA) was included in all CT scans. Participants were oriented supine within the CT gantry and both legs were simultaneously scanned. Scans included the distal femur, patella, proximal tibia, but only the proximal tibia was analyzed with this study.

CT scanning parameters included: 120 kVp tube voltage, 150 mAs tube current-time product, axial scanning plane, 0.625 mm isotropic voxel size (0.625 mm slice thickness, 0.625 mm × 0.625 mm in-plane pixel size), ~ 250 slices, ~ 60 s scan time. A standard bone kernel (BONE) was used for CT image post-processing. Effective radiation dose was ~ 0.073 mSv per scan, estimated using shareware software (CT-DOSE, National Board of Health, Herley, Denmark). For comparison, the average effective radiation dose during a transatlantic flight from Europe to North America is about 0.05 mSv [17].

Equivalent volumetric BMD (mg/cm^3^ K_2_HPO_4_) values were obtained by converting grayscale Hounsfield units (HU) to BMD using subject-specific linear regression equations developed from known densities within the QCT phantom included in each axial image (r^2^ > 0.99) [18].

### CT image analysis

#### Isolate subchondral region

**Fig. 1:**
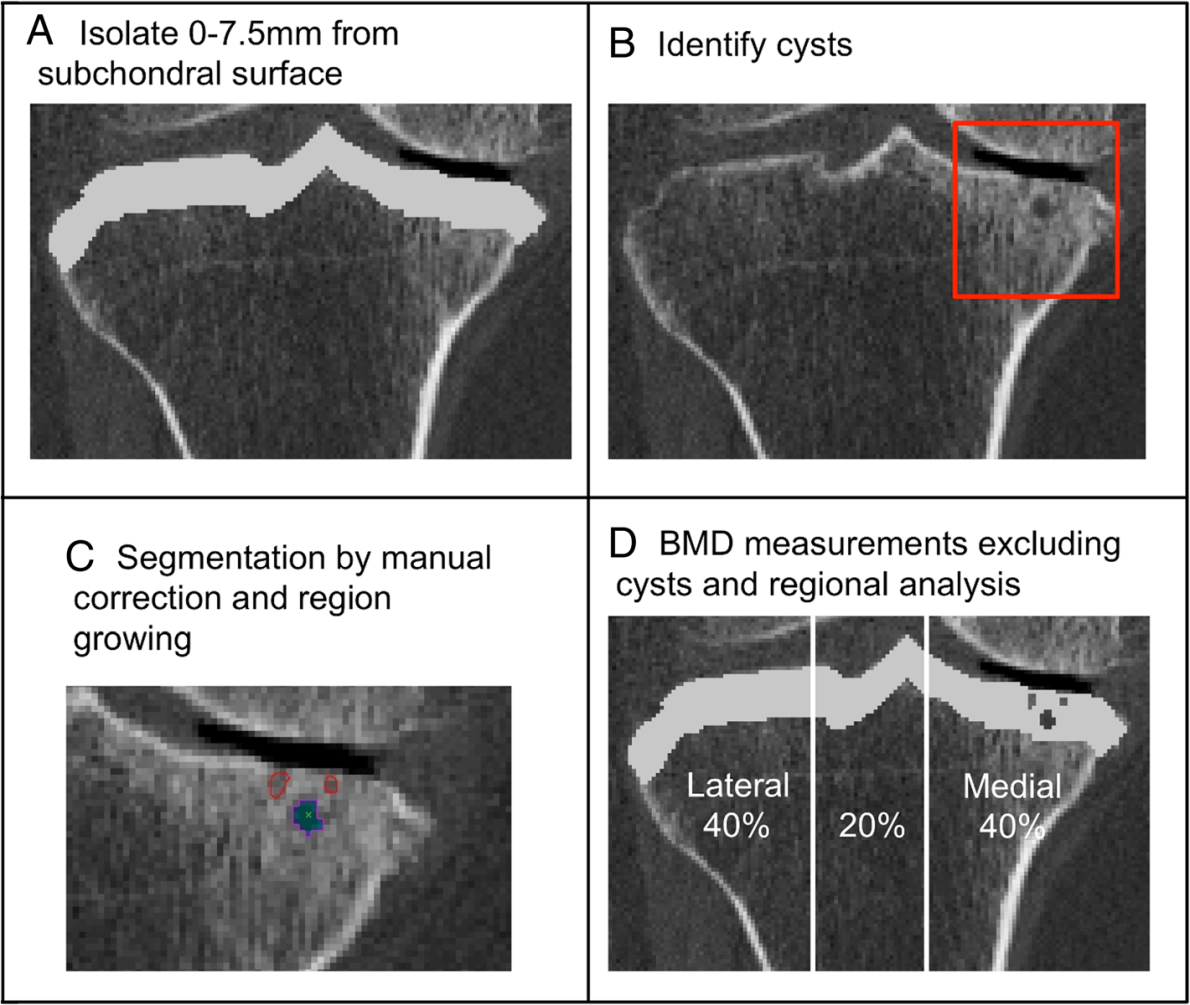
Process for identifying, isolating, and measuring cysts included isolating the subchondral region of 7.5 mm from the subchondral surface (**a**), cyst identification on CT images (**b**), and segmenting individual cysts using semi-automatic region growing for larger cysts manual segmentation for smaller cysts (**c**). Segmented image volumes were then used to measure cyst parameters, and regional BMD excluding cysts which included total, lateral, and medial regions (**d**)

We used a custom algorithm (Matlab 2016a, MathWorks, Natick, MA, USA) to isolate the subchondral region of the proximal tibia, by measuring a depth of 7.5 mm from the subchondral surface (Fig. 1a), using 3D imaged volumes of the proximal tibia from our previous work [19].

#### Cysts

Cysts were identified and manually segmented using commercial software (Analyze 10.0; Mayo Foundation, Rochester, MN, USA) and an interactive touch-screen tablet (Cintiq 21UX; Wacom, Krefeld, Germany). A single user (WDB), trained by senior researchers with guidance from an Atlas [20] and previous literature evaluating cysts using CT imaging [5], performed all segmentations. Cysts were considered as an elliptical or spherical volume of lower BMD (lower greyscale) surrounded entirely by an area of higher BMD (higher greyscale). Based upon Chiba et al. [5] and our previous studies evaluating cortical and trabecular subchondral bone [2] and the epiphyseal region tibia [19], we only considered cysts within the subchondral region at a depth of 7.5 mm from the subchondral surface (Fig. 1b). All identified cysts were segmented using semi-automatic region growing guided by subject-specific and cyst-specific threshold values defining the 50% midpoint intensity values between cyst interior and adjacent bone, similar to the half maximum height threshold (HMH) technique [21]. Smaller cysts were manually segmented (Fig. 1c). Any cyst volumes smaller than 8 voxels (1.95 mm^3^) were not included in this analysis to mitigate any errors due to noise. For cases where cysts were partially inside and partially outside the 7.5 mm depth, we only included these cysts if more than 50% of their total volume was inside the 7.5 mm depth.

We used a custom algorithm (Matlab 2016a) to measure the following cyst parameters across the subchondral region [5]: number of cysts, cyst number per total volume (number/cm^3^), cyst volume per total volume (%), total cyst volume (mm^3^), maximum cyst volume (mm^3^) and average cyst volume (mm^3^). To assess the repeatability of the cyst outcomes, a precision study was performed using recommended techniques (14 subjects scanned 3 times each = 42 image datasets) [22] whereby scans were randomized and cysts were re-identified and re-measured. Precision errors (root mean square coefficients of variation, CV%_RMS_) ranged from 0.7 to 3.6%.

### BMD

We also determined relationships between cyst parameters and subchondral BMD, excluding cysts. Segmented cyst volumes were subtracted from the previously defined subchondral volume of the proximal tibia (Fig. 1d), to determine mean BMD excluding cysts. To assess the repeatability of BMD outcomes, a precision study was performed using recommended techniques (14 subjects scanned 3 times each) [22]. Precision errors (root mean square coefficients of variation, CV%_RMS_) ranged from 0.9 to 2.4%.

### Regional analysis

Proximal tibia imaged volumes were reoriented to a neutral position where medial and lateral plateaus were approximately parallel and divided into medial, central and lateral compartments, measured by using 40% of the maximum medial-lateral axis of each respective side (Fig. 1d). We evaluated mean BMD and cyst parameters over the total, medial, and lateral proximal tibial regions.

### Statistical analysis

We report mean and standard deviation (SD) as well as median and range of cyst characteristics at total, medial, and lateral regions. As many of the measures exhibited wide and non-normal distributions, we used non-parametric Spearman’s rank correlations to determine associations between cyst parameters and participant characteristics (age, sex, BMI) and clinical characteristics of OA (medial and lateral JSN, alignment, OA severity, total WOMAC pain, nocturnal pain, BMD). We report associations between cyst characteristics and participant characteristics (age, sex, BMI) as these are known confounders for BMD [23, 24] and may be beneficial for future works evaluating proximal tibial subchondral cysts in patients with knee OA. Due to the exploratory nature of this study with multiple correlations, we report the level of significance for both *p* < 0.001 and *p* < 0.05.

## Results

Patient characteristics are found in Table 1. Cysts were present in 88% of participants (37/42). For the entire sample (*n* = 42), total cyst number varied from none (0) to up to 30 over the total proximal tibia and up to 29 in the medial compartment and 11 in the lateral compartment (Table 2). The ratio of cyst volume to tibial volume ranged from 0 to 14.8% over the total proximal tibia, and up to 24.5% in the medial compartment and 5.3% in the lateral compartments. For many cyst volume parameters, the standard deviation was similar to or greater than mean volume, indicating a large distribution in cyst volume within this sample (Table 2).

**Table 1:** Descriptive statistics for characteristics of study participants

Characteristic	Parameters
Age, years (mean ± SD)	64.1 ± 10.1
Sex (M:F)	17:25
BMI, kg/m^2^ (mean ± SD)	28.7 ± 3.7
Side (L:R)	18:24
OA Severity (KL) (Score of 0/1/2/3/4)	0/0/2/21/19
Medial OA severity (KL) (Score of 0/1/2/3/4)	3/7/7/14/11
Lateral OA severity (KL) (Score of 0/1/2/3/4)	13/18/3/6/2
WOMAC Score (score range: 0 to 20)	9.8 ± 2.9
Medial Joint Space Narrowing (JSN) (Score of 0/1/2/3)	10/6/10/14^a^
Lateral Joint Space Narrowing (JSN) (Score of 0/1/2/3)	30/5/1/4^a^
Non-weight-bearing alignment	28 varus, 6 neutral, 8 valgus
Total BMD, mg/cm^3^ K_2_HPO_4_ (mean ± SD)	279 ± 51
Medial BMD, mg/cm^3^ K_2_HPO_4_(mean ± SD)	311 ± 88
Lateral BMD, mg/cm^3^ K_2_HPO_4_(mean ± SD)	240 ± 58

^a^Data missing for 2 participants

**Table 2:** Cyst parameters for study sample (*n* = 42), mean ± SD (median, range). Cysts were present in 88% of participants (37/42). Of the 37 participants with subchondral cysts, 33 had cysts medially and 18 had cysts laterally. For these reasons, in some cases the median value is 0. Cyst parameters specific to the 37 participants with subchondral cysts can be found in Additional file 3: Table S3

	Total	Medial	Lateral
Cyst Number (Cyst.N) (#)	7.0 ± 6.7 (5.5, 0–30)	3.9 ± 5.7 (2.0, 0–29)	1.9 ± 3.0 (0.0, 0–11)
Cyst #/vol (Cyst.N/TV) (#/cm^3^)	0.3 ± 0.3 (0.3, 0–1.3)	0.4 ± 0.6 (0.2, 0–2.6)	0.2 ± 0.3 (0.0, 0–1.3)
Cyst vol/vol (Cyst.V/TV) (%)	1.3 ± 2.7 (0.0, 0–14.8)	1.9 ± 2.5 (0.0, 0–24.5)	0.2 ± 0.5 (0.0, 0–5.3)
Total cyst volume (Tot.Cyst.V) (mm^3^)	133.5 ± 245.8 (44.3, 0–1253)	98.6 ± 232.3 (15.4, 0–1156)	16.5 ± 44.1 (0.0, 0–241.0)
Maximum cyst volume (Max.Cyst.V)(mm^3^)	70.4 ± 140.6 (21.9, 0–685.8)	58.0 ± 141.5 (7.7, 0–685.8)	9.5 ± 32.5 (0.0, 0–201.9)
Average cyst volume (Avg.Cyst.V) (mm^3^)	16.7 ± 25.8 (6.6, 0–139.2)	21.9 ± 49.9 (4.9, 0–289.1)	2.6 ± 4.5 (0.0, 0–21.9)

There were no significant associations between cyst parameters and age, total WOMAC pain, or nocturnal pain (Tables 3, 4 and 5). Over the total proximal tibia, we found significant positive relationships between (a) alignment and total cyst number and (b) alignment and cyst number per tibial volume (Table 3). At the medial region, higher medial BMD was associated with greater cyst number and volume (Table 4). At the lateral region (Table 5), greater cyst number and volume were also associated with higher BMD, as well as valgus alignment, lateral KL grade, lateral JSN, sex and BMI.

**Table 3:** Correlation coefficients between cyst parameters and patient and clinical OA characteristics over the total proximal tibia. Spearman’s correlation coefficient was used for all variables. For alignment, negative relationships represent associations with varus alignment while positive relationships represent associations with valgus alignment. Significant associations are marked

	Cyst.N	Cyst.N/TV	Cyst.V/TV	Tot.Cyst.V	Max.Cyst.V	Avg.Cyst.V
Age	0.17	0.17	0.11	0.08	0.07	0.01
Sex	0.25	0.37	0.18	0.11	0.04	− 0.03
BMI	− 0.20	− 0.22	− 0.08	− 0.06	− 0.02	0.05
Alignment	0.33*	0.38*	0.19	0.16	0.09	0.02
KL	0.30	−0.07	−0.08	0.37	0.32	0.31
Total WOMAC Pain	0.25	0.29	0.16	0.15	0.07	0.05
Nocturnal Pain	0.26	0.25	0.07	0.09	−0.01	−0.09
Total BMD	0.07	0.03	0.02	0.04	0.03	0.04

*p < 0.05

**p < 0.01

**Table 4:** Correlation coefficients between cyst parameters and patient and clinical OA characteristics at the medial region. Spearman’s correlation coefficient was used for all variables. For alignment, negative relationships represent associations with varus alignment while positive relationships represent associations with valgus alignment. Significant associations are marked

	Medial Cyst.N	Medial Cyst.N/TV	Medial Cyst.V/TV	Medial Tot.Cyst.V	Medial Max.Cyst.V	Medial Avg.Cyst.V
Age	−0.03	−0.01	− 0.12	−0.13	− 0.11	−0.11
Sex	−0.03	−0.09	0.08	0.09	0.10	0.12
BMI	−0.07	−0.11	− 0.12	−0.11	− 0.10	−0.05
Alignment	−0.10	−0.09	− 0.14	−0.14	− 0.14	0.00
Medial JSN	0.08	0.06	0.13	0.13	0.12	0.02
Medial KL	0.23	0.23	0.27	0.26	0.24	0.21
Total WOMAC Pain	0.14	0.13	0.16	0.17	0.18	0.21
Nocturnal Pain	−0.09	−0.12	−0.11	−0.08	−0.08	0.00
Medial BMD	0.42*	0.41*	0.38*	0.39*	0.35*	0.33*

*p < 0.05

**p < 0.01

**Table 5:** Correlation coefficients between cyst parameters and patient and clinical OA characteristics at the lateral region. Spearman’s correlation coefficient was used for all variables. For alignment, negative relationships represent associations with varus alignment while positive relationships represent associations with valgus alignment. Significant associations are marked

	Lateral Cyst.N	Lateral Cyst.N/TV	Lateral Cyst.V/TV	Lateral Tot.Cyst.V	Lateral Max.Cyst.V	Lateral Avg.Cyst.V
Age	0.24	0.24	0.24	0.24	0.24	0.22
Sex	0.62**	0.62**	0.62**	0.62**	0.62**	0.64**
BMI	− 0.35*	− 0.35*	− 0.35*	− 0.36*	− 0.36*	− 0.34*
Alignment	0.60**	0.60**	0.61**	0.61**	0.62**	0.62**
Lateral JSN	0.66**	0.66**	0.68**	0.67**	0.68**	0.68**
Lateral KL	0.54**	0.54**	0.54**	0.54**	0.53**	0.51**
Total WOMAC Pain	0.15	0.16	0.14	0.13	0.11	0.14
Nocturnal Pain	0.24	0.25	0.23	0.23	0.22	0.20
Lateral BMD	0.48**	0.47**	0.46**	0.46**	0.45**	0.43**

*p < 0.05

**p < 0.01

From the Self-Administered Comorbidity Questionnaire, four individuals had diabetes. Exclusion of these individuals did not affect or change associations. No additional comorbidities were reported.

## Discussion

In this exploratory study using 3D in vivo QCT for analysis of proximal tibial subchondral cysts, we report cyst characteristics as well as associations between total, medial, and lateral cyst parameters and patient characteristics. At both the medial and lateral region, cyst number and volume was related to BMD. At the lateral region, cyst number and volume were also related to alignment, KL grade, JSN, sex and BMI. We found no relationships between cyst parameters and total WOMAC pain or nocturnal pain. This is the first in vivo study to use clinical QCT imaging at the knee to evaluate associations between cyst parameters and WOMAC pain, OA severity, and volumetric subchondral BMD. Exclusion of individuals with comorbidities did not affect study findings.

Our findings are consistent with prior research exploring ex vivo cyst characteristics at the hip [5] and at the tibia [7], where high cyst number per volume was also associated with high BV/TV (analogous to BMD) [5] and high trabecular thickness [7]. Cysts, which present as voids in bone, create stress concentrations [25]. High BMD is likely a response to higher stress, with the response being local bone remodelling and altered bone structure near the subchondral surface [7, 26]. High subchondral BMD, resulting from bone remodelling, may counterbalance structural instability due to cyst presence and higher stress. Surprisingly, although medial and lateral BMD were associated with medial and lateral cyst parameters, total BMD was not associated with cyst parameters over the total proximal tibia. This appears to be due to opposing findings in corresponding medial:lateral compartments (e.g., a high medial cyst number would coincide with a high medial BMD, which would coincide with a low lateral cyst number and low lateral BMD). By combining these measures, overall density was reduced but the total cyst property changed very little. This had the effect of reducing associations between total BMD and cyst properties measured over the total proximal tibia. These findings highlight the importance of assessing compartment-specific cyst properties.

Although the mechanism of cyst formation in patients with knee OA is still unknown, there are two primary hypotheses: the “bony contusion theory” [27, 28]—which proposes that excessive loading or trauma can lead to trabecular microfractures, necrotic bone, and focal bone resorption, eventually resulting in cyst development—and the “synovial fluid intrusion theory” [29, 30]—which proposes that the calcified barrier between cartilage and subchondral bone is damaged, allowing for fluid to seep into the subchondral bone, creating a fluid-filled cyst lesion.

In this work, there may be indications of each of these mechanisms in cyst formation, either independently or in combination. Over the total tibial region, the strongest associations were observed between cyst number and alignment, while at the lateral region similar associations were observed between cyst parameters and alignment as well as lateral JSN and lateral OA severity. These results suggest that cartilage degeneration may be associated with proportionally larger and more numerous cysts. Chen et al. [7] report similar findings, where subchondral cyst presence was associated with JSN and cartilage degeneration. Most likely, cyst development is a response to altered loading resulting in potential changes to bone congruence, contact forces, potentially changing load distribution through the proximal tibia [31, 32], possibly through JSN with disease progression or knee alignment. Although alignment was measured based on imaged reconstructions [3], it may be worthwhile hypothesizing why total cyst number and lateral cyst parameters were associated with valgus alignment. As the lateral compartment is predisposed to higher tibial loads in patients with valgus alignment [33, 34], patients with valgus alignment may be predisposed to higher cyst numbers and volume, even before clinical signs of OA, such as pain. However, based on this finding, it would be expected that varus alignment would be associated with medial cyst number and volume, but this relationship was not significant in our study. We recommend further studies using weight-bearing hip-to-toe radiographs to determine knee alignment to evaluate the effect of alignment on cyst characteristics and development in participants with OA.

In this study, sex and BMI were associated with cyst number and volume in the lateral compartment. To further investigate these findings related to sex, we performed comparisons to determine differences in cyst characteristics and participant characteristics between males and females (Additional file 1: Table S1 & Additional file 2: Table S2). Our results indicate that females had more and larger cysts in the lateral compartment than males (Additional file 1: Table S1). Surprisingly, of the 18 participants with lateral cysts, 17 were female and 1 was male. As lateral cyst parameters were dominated by females, an association with sex at the lateral compartment would be expected. This finding is possibly due to loading effects as females also exhibited more valgus loading (Additional file 2: Table S2), which is in line with previous research by Hvid et al. [35] and Sharma et al. [36]. Accordingly, sex and alignment may be factors to consider in future, larger scale studies evaluating proximal tibial subchondral cysts in patients with knee OA. With regards to associations between lower BMI and lateral cyst parameters, this also appears to be sex-related. Specifically, because the lateral cyst parameters were dominated by females, who had lower BMI than males (Additional file 2: Table S2), a negative association with BMI would be expected.

Interestingly, there were no associations between cyst parameters and OA severity at either the total proximal tibia or the medial compartment, but lateral OA severity was associated with all lateral cyst parameters (ρ ranged from 0.51 to 0.54, *p* < 0.01). As medial OA is more commonly reported [36–38], this work may highlight a select sub-group of patients (mostly female, mostly with valgus alignment, most with lateral cyst presence) with a tendency for more severe OA at the commonly overlooked lateral compartment. As this is a small sample of participants with severe OA, we recommend further analysis in a larger sample of patients at various stages of OA to further evaluate if this observed phenomenon is a characteristic of this sample, or generalizable to a larger sample of individuals with OA.

Although our previous work evaluating relationships between BMD and pain in this sample reported an association with high lateral focal BMD and pain [2], we found no relationships between cyst parameters and WOMAC pain. This was surprising as necrotic bone, which is found around cysts [10], is thought to contribute to pain [39]. Intra-osseous stress concentrations associated with cysts (which could approach or exceed bone’s yield strength) are also thought to lead to pain [4]. In patients with late-stage OA, cyst presence may not be related to pain in later stages of cyst development. In these cases, bone remodelling levels may be reduced or equilibrated such that the cyst structure is well formed and resistant to the effects of high stress (higher BMD found around the cysts supports this premise). We recommend further work with patients with varying stages of OA and degrees of pain to evaluate associations between cysts, BMD and pain during disease progression.

The findings of this study present various potential clinical impacts. Using clinical QCT we were able to measure relationships between cyst parameters (number, size, volume) and participant characteristics in vivo as had previously only been done ex vivo using non-clinical imaging tools such as micro-CT [7] and HR-QCT [5]. This presents a promising clinical QCT-based technique for monitoring similar cyst parameters and regional BMD in a clinical sample and the ability to incorporate clinical symptoms such as pain. Clinical studies using MRI-based techniques present that cyst presence may [8, 40] or may not [12, 41, 42] be related to pain in patients with OA. Although these studies show that it is possible to distinguish cysts and cyst-like lesions using MR, resolution limitations inhibit the ability to distinguish and measure smaller subchondral cysts. QCT provides higher resolution, as well as the ability to easily quantify cyst-adjacent BMD. This work further demonstrates the complexities of cyst presence in relation to pain in patients with OA, especially in late-stage OA, but may provide a comprehensive technique able to distinguish cysts, as well as regional or localized BMD in vivo.

This study has certain limitations to consider. First, our measurements use a larger voxel size than ex vivo approaches such as micro-CT and may ignore smaller cysts. It was difficult to reliably quantify any cysts smaller than 8 voxels (2x2x2 voxels or 1.95mm^3^). It was also challenging to differentiate between small cysts and surrounding bone. Second, it was challenging to determine individual cysts with adjacent large cysts that would sometimes connect with one another in some participants. In these cases, these were counted as a single large cyst, but could also be regarded as multiple smaller cysts, which could have merged into a large cyst-like void. This could account for multiple large cysts within our study. Third, our alignment measurement was a custom in-house approach using CT reconstructions and relative joint space widths to approximate mechanical alignment. Although this technique has not been validated against full-limb radiographs, in these patients with late OA malalignment was evident with values ranging from − 18° to 8° from neutral position [3]. Fourth, as this was an in vivo study, we did not include biochemical or histological analysis, and are thus uncertain of cyst genesis or development. Fifth, our sample has various limitations including participants at late stage of OA severity and pain measurement, as well as wide and non-normal variable distribution. Our sample represented patients at late stages of OA and findings may not be applicable to patients with early OA. Also, as this sample did not include healthy control subjects, it is difficult to determine if observed associations are specific to individuals with OA, or pertain to the healthy population as well. WOMAC pain severity and assessment was based on the entire knee joint, including all joint surfaces (tibiofemoral and patellofemoral) and tissues (e.g., bone, menisci, synovium), and it is uncertain if pain originated within the proximal tibial bony structure, other tissues, or a combination of tissues. It is uncertain if wide and non-normal distribution of cyst characteristics may be an element specific to this small sample of participants with severe OA and high amounts of pain, or if this is common over a larger sample with more varied pain or disease progression. Given that cyst parameters of the proximal femur reported by Chiba et al. also exhibited non-normal distributions [5], we believe non-normal distributions are common. However, further prospective evidence from participants at varying initial stages of OA and OA-related pain is needed to complement these preliminary findings. Such research will help to clarify the relationship between cyst parameters and patient characteristics, as well as the role of subchondral cysts in knee OA and OA-related pain.

## Conclusions

In this exploratory study, we used clinical QCT to analyze subchondral cysts at the proximal tibia of OA patients to determine relationships between subchondral cyst parameters and clinical characteristics of OA. There was a large range in cyst number and volume in our sample, suggesting that cyst development and progression may vary from patient to patient. Greater cyst number and volume were associated with higher BMD at both the medial and lateral compartments of the proximal tibia. At the lateral region, cyst number and volume were also strongly associated with several patient characteristics including joint alignment, sex, lateral OA severity and lateral JSN, suggesting that there may be disease-associated changes in tibial loading distribution leading to cyst development and further joint deterioration. We found no associations between cyst number or cyst volume with OA-related knee pain in patients with late-stage OA. As such, it may be further worthwhile to explore other bone-related outcomes (e.g., BMLs, BMD), when investigating which structures are associated with pain.

## Additional files

Additional file 1: **Table S1** Mann-Whitney U tests determining differences in cyst characteristics between male and female participants. We report medians and ranges. Significant differences (*p* < 0.05) are bolded. With regards to why no results are provided for Males at the lateral compartment, this is because only 1 male had lateral cysts. (DOCX 17 kb)

Additional file 2: **Table S2** Differences in patient characteristics between males and females. Independent samples t-tests were used for continuous variables and Chi-squared tests were used for categorical variables (noted in italics). Significant differences are bolded. (DOCX 18 kb)

Additional file 3: **Table S3** Cyst parameters from participants only with cysts specific to each compartment, mean ± SD (median, range). (DOCX 19 kb)

## Abbreviations

BMD: Bone mineral density
BML: Bone marrow lesion
BV/TV: Bone volume per total volume
CT: Computed tomography
CV%: Coefficients of variation
FE: Finite element
HMH: Half-maximum height
HR-QCT: High-resolution quantitative computed tomography
HU: Hounsfield units
JSN: Joint space narrowing
KL: Kelgren-Lawrence score
MRI: Magnetic resonance imaging
OA: Osteoarthritis
QCT: Quantitative computed tomography
WOMAC: Western Ontario and McMaster Universities Arthritis Index
