# Supplementary material for: Knee osteoarthritis patients with more subchondral cysts have altered tibial subchondral bone mineral density
Source: BMC Musculoskelet Disord. 2019 Jan 5;20:14. doi: 10.1186/s12891-018-2388-9 (PMC6320646; doi:10.1186/s12891-018-2388-9)
Supplement: Supplementary file 1 — Table S1 Mann-Whitney U tests determining differences in cyst characteristics between male and female participants. We report medians and ranges. Significant differences (p < 0.05) are bolded. With regards to why no results are provided for Males at the lateral compartment, this is because only 1 male had lateral cysts. (DOCX 17 kb) [file 12891_2018_2388_MOESM1_ESM.docx]

Supplemental Table 1. Mann-Whitney U tests determining differences in cyst characteristics between male and female participants. We report medians and ranges. Significant differences (*p*<0.05) are bolded. With regards to why no results are provided for Males at the lateral compartment, this is because only 1 male had lateral cysts.

|  |  | Sex | | | | *p* |
| --- | --- | --- | --- | --- | --- | --- |
|  |  | Males | | Females | |  |
|  |  | Median | Range | Median | Range |  |
| Total | |  |  |  |  |  |
|  | Cyst.N | 7 | 0 - 30 | 8 | 0 - 28 | 0.177 |
|  | Cyst.N/TV | 0.21 | 0.0 - 1.0 | 0.34 | 0.0 - 1.3 | 0.018 |
|  | Cyst.V/TV | 0.2 | 0.0 - 6.4 | 0.5 | 0.0 - 14.8 | 0.282 |
|  | Tot.Cyst.V | 68.8 | 0.0 - 853 | 83.7 | 0.0 - 1253 | 0.434 |
|  | Max.Cyst.V | 28.5 | 0.0 - 517 | 27.1 | 0.0 - 685.8 | 0.818 |
|  | Avg.Cyst.V | 9.5 | 0.0 - 60.9 | 7.8 | 0.0 - 139.2 | 0.838 |
| Medial | |  |  |  |  |  |
|  | Cyst.N | 9 | 0.0 - 29 | 3 | 0 - 17 | 0.745 |
|  | Cyst.N/TV | 0.8 | 0.0 - 2.6 | 0.4 | 0.0 - 2.1 | 0.709 |
|  | Cyst.V/TV | 0.3 | 0.0 - 6.7 | 0.1 | 0.0 - 13.7 | 0.887 |
|  | Tot.Cyst.V | 55.7 | 0.0 - 852.8 | 48.8 | 0.0 - 1156 | 0.728 |
|  | Max.Cyst.V | 17.8 | 0.0 - 517.1 | 11.9 | 0.0 - 685.8 | 0.827 |
|  | Avg.Cyst.V | 7.8 | 0.0 - 60.9 | 5.5 | 0.0 - 289.1 | 0.817 |
| Lateral | |  |  |  |  |  |
|  | Cyst.N | 0 | 0 - 1 | 5 | 0 - 11 | <0.001 |
|  | Cyst.N/TV | 0 | 0.0 - 0.1 | 0.5 | 0.0 - 1.3 | <0.001 |
|  | Cyst.V/TV | 0 | 0.0 - 0.01 | 0.0 | 0.0 - 2.9 | <0.001 |
|  | Tot.Cyst.V | 0 | 0.0 - 2.0 | 26.4 | 0.0 - 241.0 | <0.001 |
|  | Max.Cyst.V | 0 | 0.0 - 2.0 | 10.5 | 0.0 - 201.9 | <0.001 |
|  | Avg.Cyst.V | 0 | 0.0 - 2.0 | 5.1 | 0.0 - 21.9 | <0.001 |
