# Supplementary material for: Knee osteoarthritis patients with more subchondral cysts have altered tibial subchondral bone mineral density
Source: BMC Musculoskelet Disord. 2019 Jan 5;20:14. doi: 10.1186/s12891-018-2388-9 (PMC6320646; doi:10.1186/s12891-018-2388-9)
Supplement: Supplementary file 2 — Table S2 Differences in patient characteristics between males and females. Independent samples t-tests were used for continuous variables and Chi-squared tests were used for categorical variables (noted in italics). Significant differences are bolded. (DOCX 18 kb) [file 12891_2018_2388_MOESM2_ESM.docx]

Supplemental Table 2. Differences in patient characteristics between males and females. Independent samples t-tests were used for continuous variables and Chi-squared tests were used for categorical variables (noted in italics). Significant differences are bolded.

| Characteristic | Males (n=17) | Females (n=25) | *p* |
| --- | --- | --- | --- |
| Age, years  (mean ± SD) | 63.5 ± 10.7 | 64.5 ± 9.9 | 0.745 |
| BMI, kg/m^2^  (mean ± SD) | 29.8 ± 2.5 | 27.9 ± 4.2 | 0.090 |
| *Side (L:R)* | 7:10 | 11:14 | 0.856 |
| *OA Severity (KL)*  *(Score of 0/1/2/3/4)* | 0/0/0/10/7 | 0/0/2/11/12 | 0.385 |
| *Medial OA Severity (KL)*  *(Score of 0/1/2/3/4)* | 0/0/4/8/5 | 3/7/3/6/6 | 0.053 |
| *Lateral OA Severity (KL)*  *(Score of 0/1/2/3/4)* | 7/10/0/0/0 | 6/8/3/6/2 | **0.038** |
| WOMAC Score  (mean ± SD) | 9.2 ± 2.6 | 9.2 ± 2.6 | 0.274 |
| *Nocturnal pain*  *(Score of 0/1/2/3/4)* | 3/4/7/2/1 | 6/4/9/6/0 | 0.587 |
| *Medial Joint Space Narrowing (JSN)**  *(Score of 0/1/2/3)* | 0/3/7/7 | 10/3/3/7 | **0.012** |
| *Lateral Joint Space Narrowing (JSN)**  *(Score of 0/1/2/3)* | 17/0/0/0 | 13/5/1/4 | **0.020** |
| *Non-weight-bearing alignment*  *(varus/neutral/valgus)* | 15/2/0 | 13/4/8 | **0.023** |
| Total BMD, mg/cm^3^ K_2_HPO_4_  (mean ± SD) | 294 ± 37 | 270 ± 56 | 0.164 |
| Lateral BMD, mg/cm^3^ K_2_HPO_4_  (mean ± SD) | 224 ± 36 | 249 ± 66 | 0.190 |
| Medial BMD, mg/cm^3^ K_2_HPO_4_  (mean ± SD) | 313 ± 132 | 284 ± 94 | 0.419 |
| *Data missing for 2 participants | | | |
