# Supplementary material for: Knee osteoarthritis patients with more subchondral cysts have altered tibial subchondral bone mineral density
Source: BMC Musculoskelet Disord. 2019 Jan 5;20:14. doi: 10.1186/s12891-018-2388-9 (PMC6320646; doi:10.1186/s12891-018-2388-9)
Supplement: Supplementary file 3 — Table S3 Cyst parameters from participants only with cysts specific to each compartment, mean ± SD (median, range). (DOCX 19 kb) [file 12891_2018_2388_MOESM3_ESM.docx]

Supplemental Table 3. Cyst parameters from participants only with cysts specific to each compartment, mean ± SD (median, range).

|  | Total (n=37) | Medial (n=33) | Lateral (n=18) |
| --- | --- | --- | --- |
| Cyst Number (Cyst.N) (#) | 7.8±6.6 (7.0, 1-30) | 34.9±6.0 (2.0, 1-29) | 4.3±3.3 (4.5, 1-11) |
| Cyst #/vol (Cyst.N/TV) (#/cm^3^) | 0.3±0.3 (0.3, 0-1.3) | 0.5±0.6 (0.2, 0.8-2.6) | 0.5±0.4 (0.4, 0.1-1.3) |
| Cyst vol/vol (Cyst.V/TV) (%) | 1.5±2.8 (0.0, 0-14.8) | 2.0±5.1 (0.3, 0.0-24.5) | 0.7±1.4 (0.2, 0.0-5.3) |
| Total cyst volume (Tot.Cyst.V) (mm^3^) | 148.1±254.4 (62.3, 2.0-1253) | 125.5±256.2 (31.7, 2.0-1156) | 38.5±61.5 (18.0, 2.0-241.0) |
| Maximum cyst volume (Max.Cyst.V)(mm^3^) | 77.8±146.0 (27.2, 2.0-685.8) | 73.9±156.3 (12.0, 2.0-685.8) | 22.1±47.4 (7.1, 2.0-201.9) |
| Average cyst volume (Avg.Cyst.V) (mm^3^) | 18.5±26.6 (8.7, 2.0-139.2) | 27.8±54.9 (6.6, 2.0-289.1) | 6.2±5.2 (4.4, 2.0-21.9) |
